# Polyploidy Improves Photosynthesis Regulation within the *Ranunculus auricomus* Complex (Ranunculaceae)

**DOI:** 10.3390/biology10080811

**Published:** 2021-08-21

**Authors:** Fuad Bahrul Ulum, Franz Hadacek, Elvira Hörandl

**Affiliations:** 1 Department of Systematics, Biodiversity and Evolution of Plants, Albrecht-von-Haller Institute for Plant Sciences, University of Göttingen, 37073 Göttingen, Germany; fuadbahrul.ulum@stud.uni-goettingen.de; 2 Georg-August University School of Science (GAUSS), University of Göttingen, 37073 Göttingen, Germany; 3 Biology Department, Faculty of Mathematics and Sciences, Jember University, Jember 68121, Indonesia; 4 Department of Plant Biochemistry, Albrecht-von-Haller Institute for Plant Sciences, University of Göttingen, 37077 Göttingen, Germany; franz.hadacek@biologie.uni-goettingen.de

**Keywords:** apomixis, Kautsky curve, OJIP, quantum yield, light stress, photochemical quenching, non-photochemical quenching, habitat adaptation

## Abstract

**Simple Summary:**

Genome duplication or multiplication, polyploidy, has contributed substantially to the evolutionary success of plants. Polyploidy is often connected to a higher resilience to environmental stress. We have chosen the goldilocks, the *Ranunculus auricomus* complex, to study effects of light stress. In this species complex, diploid (2x), tetraploid (4x), and hexaploid (6x) cytotypes occur in Central Europe in both shaded and sun-exposed habitats. In this study, we exposed them to different photoperiods in climate growth chambers to explore how the efficiency of photosynthesis varied between the various ploidies (2x, 4x, and 6x). We used fluorescence experiments exploring the proportion of light that is captured for photosynthesis and the resulting energy fluxes. In addition, quenching coefficients can be calculated that inform about the capability of a plant to deal with excess light. We found that the polyploids can quench excess light better, which concurs with their adaptation to open habitats and their predominantly asexual mode of reproduction that is probably favored by low stress levels in the reproductive tissues.

**Abstract:**

Polyploidy has substantially contributed to successful plant evolution, and is often connected to a higher resilience to environmental stress. We test the hypothesis that polyploids tolerate light stress better than diploids. The *Ranunculus auricomus* complex comprises diploid (2x), tetraploid (4x), and hexaploid (6x) cytotypes, the former of which occur in shaded habitats and the latter more in open, sun-exposed habitats in Central Europe. In this study, we experimentally explored the effects of ploidy and photoperiod extension on the efficiency of photosystem II in the three cytotypes in climate growth chambers. Quantum yields and various coefficients that can be calculated from light curve, Kautsky curve, and fluorescent transient OJIP experiments provided support for the hypothesis that, in comparison to diploids, the improved regulation of excess light by more efficient photochemical and non-chemical quenching in polyploids might have facilitated the adaptation to unshaded habitats. We suggest how lower stress levels in reproductive tissues of polyploids might have favored asexual reproduction.

## 1. Introduction

Polyploidy, whole-genome multiplication, denotes the presence of double or multiple chromosome sets by either genome doubling in a single species (autopolyploidy), or by hybridization of two species with associated genome doubling (allopolyploidy) [1]. Polyploidy enhances stress tolerance in response to drastic environmental changes by enabling more extensive adaptations as consequences of gene and genome duplication [2] and acts as a driver of evolution and speciation in plants [3]. Compared to their diploid progenitor, polyploids exhibit better stress resistance [2]. For example, they are able to increase abscisic acid (ABA) signaling under drought conditions [4], alter volatile profiles and photosynthesis performance under cold stress [5], and increase non-photochemical quenching (NPQ) and xanthophyll production in light stress [6]. Polyploid plants can perform photosynthesis more efficiently [7] because they have larger mesophyll cells containing more chloroplasts, and thus more chlorophyll and expressed ribulose-1,5-bisphosphate-carboxylase/-oxygenase (RuBisCo) in comparison to their diploid relatives [8,9]. Moreover, the higher diversity in their genomes, transcriptomes, and metabolomes of polyploids may contribute to their higher resilience to environmental stress [10].

In plants, photoperiod extension can induce flower meristem development [11], enhance photosynthesis efficiency [12,13], growth [14], and metabolite biosynthesis [15], as a result of adjusting the circadean oscillator [16]. In a survey of 23 tree species, photoperiod affected photosynthesis more profoundly than temperature [12].

Light stress in plants occurs whenever light absorption in leaves exceeds those levels that can be utilized as energy and those that can be buffered by dissipation capabilities [17]. Chloroplasts, besides mitochondria and peroxisomes, represent the major source of reactive oxygen species (ROS) that can damage cell components if their concentrations rise above levels that are used for signaling [18]. When photodamage exceeds the antioxidant repair capacity, photosystem II (PSII) is downregulated (photoinhibition) [19]. Plants can avoid this situation by repartitioning the energy of the absorbed light between photochemistry and energy dissipating pathways as a photoprotective mechanism, among which we differentiate photochemical (PQ) and non-photochemical quenching (NPQ) processes [17,20].

Among plant organs, the reproductive parts are most sensitive to stress, e.g., moderate temperature stress can reduce seed set [21]. Water, temperature, and light stress can hamper male gamete and female ovule development [22]. The development of apomixis, asexual reproduction via seeds [23], can also be affected by stress. Apomixis is usually facultative, which means that the same plant can produce both sexual and apomictic seeds [23]. Frequencies of sexual reproduction in facultative apomictic plants increased after stress, e.g., in *Boechera* [24], *Ranunculus* [25,26], *Eragrostis* [27], and *Paspalum* [28]. The putative background for this phenomenon is that increased oxidative stress in ovules triggers initiation of meiosis [24,29]. The genetic regulation of apomixis, however, is complex (Schmidt 2020), and differs from the cytological processes of microsporogenesis and formation of unreduced male gametes [30,31]. Apomeiosis, the production of unreduced embryo sacs [23], is the key developmental step in gametophytic apomixis in the *Ranunculus auricomus* complex [32]. This polyploid complex is a well-established model system for expression of facultative apomixis [31,32,33,34,35] and for evolution of polyploid cytotypes [36,37,38,39]. The polyploid cytotypes are evolutionarily young (less than 100,000 years old [40]) and exhibit a low genetic divergence [26]. The *Ranunculus auricomus* complex comprises diploid sexual and tetraploid and hexaploid facultative apomictic, aposporous, and pseudogamous lineages and they develop their flowers under short day conditions (nights ≥ 12 h) [32,39]. In facultative apomictic polyploids, higher stress levels increased proportions of meiotic compared to apomeiotic ovules [25,26,29]. Congruently, a recent study on the *Ranunculus auricomus* complex over a large geographical area in Europe revealed that, among many climatic parameters, light intensity was positively correlated to the distribution of sexual reproduction [39].

The reproduction mode of the same three cytotypes that were used in this study have been investigated previously in terms of reproduction mode development in different photoperiods [25,26]. Extended photoperiods reduced formation of asexual ovules in favor of meiotic ones. We hypothesize that lower stress levels in polyploids favor apomictic reproduction. The major aim of the present study was to investigate how ploidy level and extended photoperiod affect photosynthesis efficiency and to explore if these insights provide hypotheses in efforts to explain the observed shifts in ovule type formation.

Three cytotypes of the *R. auricomus* complex (2x, 4x, and 6x [32]), with different ecological amplitudes, were exposed to the more or less adapted (10 h) and a moderately extended photoperiod (16.5 h) [25]. We examined the photosynthetic efficiency by performing various chlorophyll fluorescence experiments, quantum yield in light-adapted plants and dark-adapted plants, relative electron transport rates (*rETR*) during increasing actinic light (light curves), fluorescence induction in actinic light and darkness (Kautsky curves), and fast fluorescence transients (OJIP) [41,42,43]. Fluorescence coefficients that were obtained from the Kautsky curves allowed the calculation of a number of coefficients that quantify photosystem II quenching processes, PQ and NPQ [44]. The OJIP analysis provides coefficients that inform about PSII energy fluxes and general performance [45,46]. In terms of exploring photosynthesis efficiency of different ploidies and the effects of an extended photoperiod on them, to the knowledge of the authors, a comparable comprehensive range of fluorescence experiments has not been performed previously. The expected insights are aimed to contribute to a better understanding of to what extent photosynthetic performance and stress response can contribute to the formation of apomictic seeds in plants, a trait to which plant breeders pay still a lot of attention as one mechanism to fix vigorous hybrid genotypes over generations [47,48]. Since many crop plants are polyploids, we further contribute to the understanding of photosynthesis performance of different cytotypes.

## 2. Materials and Methods

### 2.1. Plant Material

*Ranunculus auricomus* plants comprised the same individuals as in a previously published study [26]. Table 1 provides an overview and Table S1 shows which experiments have been performed on which individuals. The sampling covers the whole morphological diversity of leaf shape within the complex, as the diploid cytotype forms both divided and undivided basal leaves [49], the tetraploids have mostly divided leaves [36], while both hexaploid clones have mostly undivided leaves [38]. We did not observe any effects of light treatments on morphological traits. All seedlings developed under equal conditions outdoors in the Old Botanical Garden of the University of Goettingen. At all ploidy levels, plants represented closely related hybrid genotypes [26].

### 2.2. Photoperiod Experiments in Climate Chambers

Growth conditions for control and stress treatments were optimized in a previous study [25]. The climatic chambers were set at a temperature of 18 °C, 60% humidity, and an average light intensity of about 250 µmol photons m^−2^ s^−1^. The photoperiod spanned 10 h for control and 16.5 h for light stress treatment. Consequently, the plants received 9.0 mol m^−2^ d^−1^ and 14.8 mol m^−2^ d^−1^, respectively.

### 2.3. Photosynthesis

We analyzed the effect of extended photoperiod on photosynthesis efficiency as a proxy of stress conditions. All photosynthesis analyses were performed on 3–11 plants per ploidy level and treatment. A fully developed basal leaf (upper side) that supported the inflorescence was chosen for the measurement. Fluorescence analyses started in the first weeks after sprouting in March 2019 when plants produced flower buds. Flower buds are covered by green sepals as photosynthetic tissue, of which no measurements were possible with the available equipment. Basal leaves had to suffice under assumptions that effects on photosynthesis were comparable in both leaf types.

The photosynthesis performances were observed by measurement of chlorophyll fluorescent intensity with a PAM fluorometer, PAR-FluorPen FP 110 LM/S (Photon Systems Instruments, Drásov, Czech Republic). Table S2 shows the used coefficients and their calculations on the basis of the obtained fluorescence data. By using the preprogrammed device protocol, first, we measured the leaves without pre-dark adaptation to record the PSII potential quantum yield y (*ϕ_PSII_*). Then, plants were dark-adapted for at least 30 min for further parameter measurements, i.e., a light curve (LC) to determine the relative electron transport rate (*rETR*), Kautsky curve fluorescence induction decay (KC) to explore photochemical (PQ) and non-photochemical quenching (NPQ), and fast fluorescence transient curve (OJIP). Maximum quantum efficiency of PSII (*ϕ_max_*) was determined in all dark-adapted experiments (LC, KC, and OJIP).

#### 2.3.1. Light Curves (LC)

After dark adaptation, the experiment was started with an initial saturating super pulse in the dark, and subsequently, similar pulses were applied during an actinic light phase with increasing intensity, 10, 20, 50, 100, 300, and 500 µmol photons m^−2^ s^−1^. Higher light intensities were not used due to a strong intensity decrease at 500 µmol photons m^−2^ s^−1^ actinic light. The measuring pulse was set to 0.09 µmol photons m^−2^ s^−1^, the saturating and super pulses to 2400 µmol photons m^−2^ s^−1^. The coefficient *rETR* was calculated as shown in Table S2.

#### 2.3.2. Kautsky Curve Fluorescence Induction Decay (KC)

The measuring and super pulses were the same as in the light curve analysis (LC). The actinic light was set to 300 µmol photons m^−2^ s^−1^. After the initial saturating super pulse, actinic light was switched on and lasted for 1 min. Within this time period, five additional super pulses were applied, the first one after 7 s and the following in 12 s intervals. The subsequent dark period lasted 88 s, during which three super pulses were applied, the first one after 11 s and the following in 26 s intervals. The fluorescence parameters *F_0_*, *F_M_*, *F_M_′*, *F(t)*, *F_0_″*, and *F_M_″* (Figure S1) allowed calculating a number of coefficients (Table S2), among which non-photochemical quenching (*NPQ*), energy-dependent non-photochemical quenching coefficient (*q_E_*), and photoinhibitory photochemical quenching coefficient (*q_I_*) informed about non-photochemical quenching (NPQ) and photochemical quenching (*PQ*) together with the photochemical quenching coefficients *q_P_* and *q_L_* about photochemical quenching (for detailed explanations, see Table S2).

#### 2.3.3. Fast Fluorescence Transient Analysis (OJIP)

OJIP (alternatively O-J-I-P) experiments followed Strasser et al. [43]. The OJIP curve was induced by a pulse of red light of 3000 µmol photons m^−2^ s^−1^. The relative fluorescence intensity of the OJIP curve at time points O, J, I, and P, 0.5, 2, 30, and 1000 ms, respectively (*F_0_*, *F_J_*, *F_I_*, and *F_P_*), was determined after dark adaptation. Several OJIP-specific coefficients can be calculated (Table S2). Additional individuals had to be included as the number of available remaining basal leaves from the hitherto investigated individuals was insufficient (Table S1).

### 2.4. Statistical Analysis

All statistical analyses were conducted with R (version 4.0.2 for Windows, R Foundation for Statistical Computing, [50]). Data handling and visualization were performed using the packages *dplyr* [51], *tidyr* [52], and *ggplot2* [53]. Boxplots were created with *ggpubr* [54]. Differences in the photoperiods’ effect on the photosynthetic performance of the different ploidies in the two photoperiods were explored with ANOVA and a 95% Tukey or 95% Duncan multiple range test (*glmmTMB* [55]).

## 3. Results

### 3.1. Cytotype 6x_35 Showed Lower PSII Potential Quantum Yield (ϕ_PSII_) in Both Photoperiods

**Figure 1:**
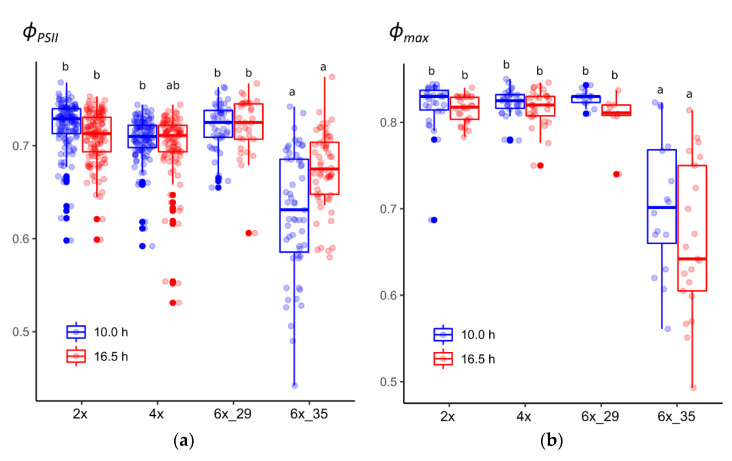
Quantum yields of *R. auricomus* cytotypes exposed to two different photoperiods: (**a**) potential quantum yield, *ϕ_PSII_*, from light-adapted plants; (**b**) maximum quantum yield, *ϕ_max_*, from dark-adapted plants. For summary statistics, see Table S3, letters represent 95% Tukey. Boxplots show the 25th, median, and 75th percentile range, and jitter plots represent the exact data distribution.

The extended photoperiod did not affect the light-adapted *ϕ_PSII_* in the tested cytotypes, with clone 6x_35 being the exception that showed significantly lower *ϕ_PSII_* values (Figure 1a). Initially, both hexaploid cytotypes were regarded as sufficiently uniform to contribute to the investigated 6x cytotypes. The high variability in the experimental data and the calculated coefficients caused us to re-evaluate to what extent each of the two clones, 6x_29 and 6x_35, contributed to the observed variation. One clone, 6x_29, was found to yield rather homogenous data compared to the other cytotypes. The other clone, 6x_35, by contrast, deviated by predominantly yielding lower *ϕ_PSII_* values. Consequently, and in contrast to the original experimental plan, the two clones were treated separately in attempts not to hamper statistical data evaluation by inhomogeneous sample groups.

### 3.2. Cytotype 6x_35 Showed Lower PSII Maximum Quantum Efficiency (ϕ_max_) in Both Photoperiods

Likewise, the extended photoperiod did not affect the dark-adapted maximum quantum yield of PSII (*ϕ_max_*) with the only exception again being clone 6x_35 that differed significantly from the others (Figure 1b). Interestingly, after dark adaptation, 6x_35 plants that had been exposed to the extended photoperiod showed a lower median than in the case of *ϕ_PSII_*, in which the median of the 10 h-exposed plants was lower.

### 3.3. A Visual Comparison of Fluorescence Experimental Data Hinted at Subtle Differences between Ploidies and Photoperiods

**Figure 2:**
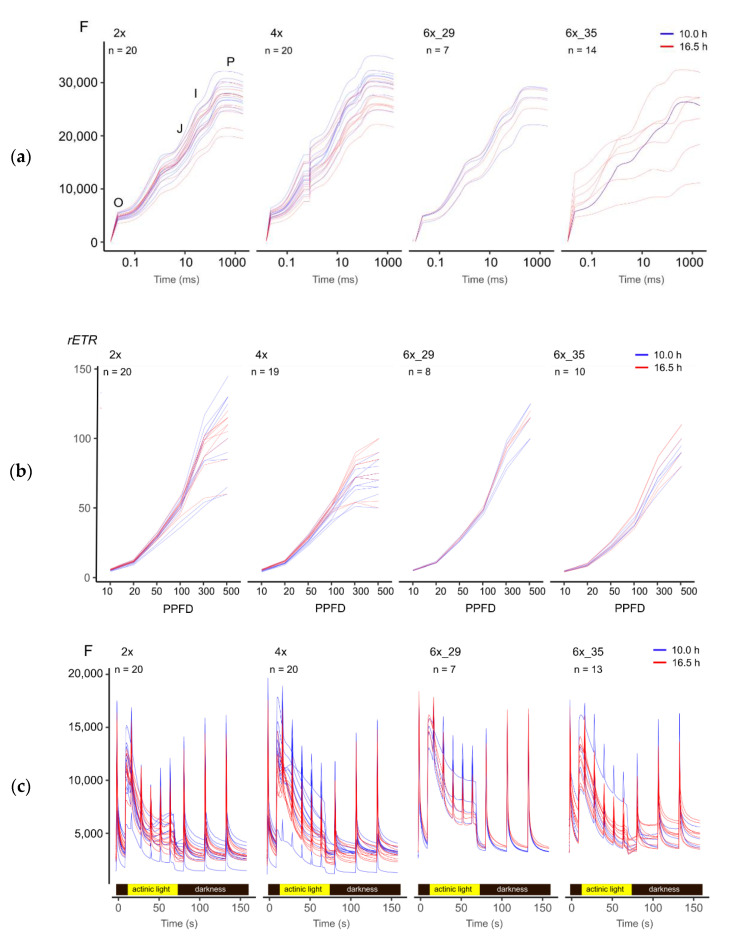
Visual overview of fluorescence experiments of *R. auricomus* cytotypes exposed to two different photoperiods: (**a**) fluorescent transient (OJIP) (**b**) light curve (LC) with increasing photosynthetic photon flux density (PPFD, µmol photons m^−1^ s^−1^), relative electron transport rate (*rETR*); (**c**) Kautsky curves (KC).

Figure 2 provides a graphical overview of the various fluorescence-based experiments. The OJIP transient curves represent a meticulous analysis of a single super pulse after dark adaptation (Figure 2a). Relative electron transport rates (*rETR*) were calculated from the LC experiment that explored the effect of increasing actinic light intensity (PPFD) on *ϕ_PSII_* (Figure 2b). Kautsky curves monitor the decay of fluorescence induction in actinic light and a subsequent dark period (Figure 2c).

**Figure 3:**
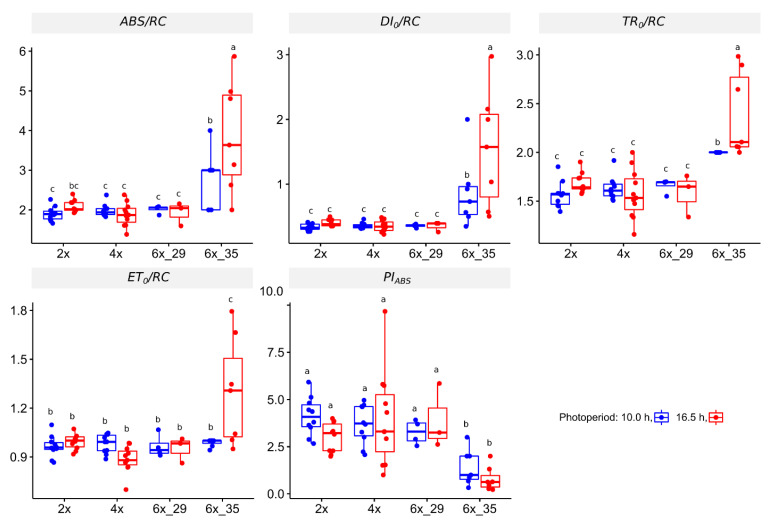
Coefficients calculated from fluorescent transient (OJIP) analysis of *R. auricomus* cytotypes exposed to two different photoperiods; absorption energy flux per PSII center (*ABS/RC*), dissipated energy fl. (*DI*_0_*/RC*); trapper energy fl. (*TR*_0_*/RC*); electron transport fl. (*ET*_0_*/RC*); performance index on absorption basis related to PSII activity (*PI_ABS_*); for summary statistics, see Table S4, letters represent 95% Duncan. Boxplots show the 25th, median, and 75th percentile range, and jitter plots represent the exact data distribution.

Albeit revealing no specific insights, Figure 2 points to the subtle differences between ploidies and photoperiods that merited further exploration. For example, (1) OJIP curves of the 6x_35 varied much more than the other cytotypes (Figure 2a); (2) the light curves of the tetraploids differed clearly from the other cytotypes (Figure 2b), and (3) different photoperiods tended to cause some differentiation in the polyploid Kautsky curves but not those of the diploid cytotypes (Figure 2c).

### 3.4. Photoperiod and Ploidy Effects

#### 3.4.1. OJIP Experiment

This OJIP experiment focuses just on the effect of one super pulse on chlorophyll fluorescence after dark adaptation, albeit in a very meticulous way. Figure 3 presents a number of coefficients that quantify specific energy fluxes, *ABS/RC, DI*_0_*/RC, ET0/RC*, and *TR_0_/RC*, as well as a general performance index, *PI_ABS_*. Only cytotype 6x_35 differed from the others investigated. This became especially evident in the case of the photoperiod extension that caused significant increases in the energy flux coefficients, such as absorption flux per PS II reaction center (ABS/RC), dissipated energy (*DI*_0_*/RC*), electron transport flux (*ET*_0_*/RC*), and trapped energy per reaction center (TR_0_/RC). The performance index PI*_ABS_* decreased.

#### 3.4.2. Light Curve (LC) Experiment

**Figure 4:**
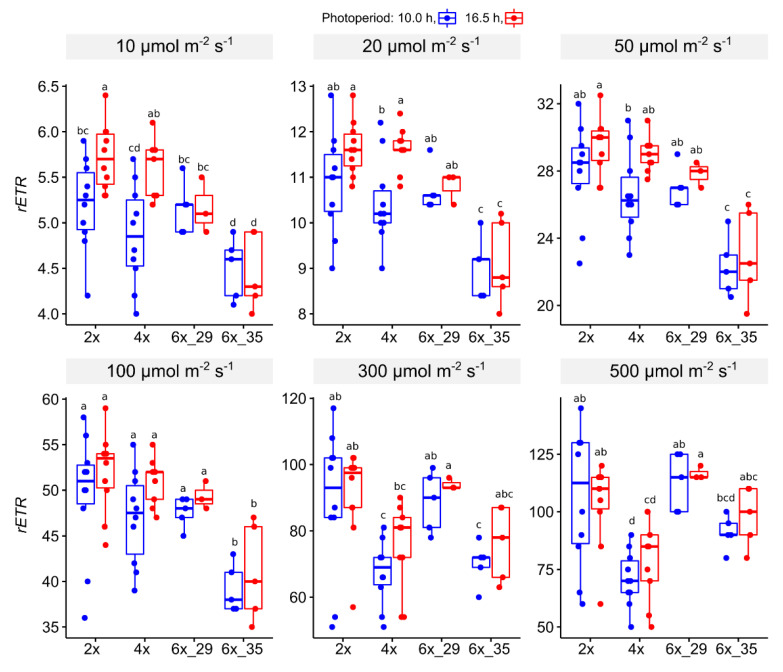
Relative electron transport rate (rETR) from light curve (LC) experiments of *R. auricomus* cytotypes exposed to two different photoperiods in increasing actinic light PPFD; for summary statistics, see Table S5, letters represent 95% Duncan. Boxplots show the 25th, median, and 75th percentile range, and jitter plots represent the exact data distribution.

At lower PPFDs, 10–100 µmol m^−1^ s^−1^, the 6x_35 clone showed the lowest rETR whilst the other ploidies did not differ (Figure 4). At values ≥ 300 µmol m^−2^ s^−1^, a remarkable change occurred, in which the 4x plants took over in showing the lowest values.

In terms of photoperiod extension, significant differences only appeared in lower PPFDs < 50 µmol m^−1^ s^−1^. Plants that were exposed to the longer photoperiod, 16.5 h, showed higher *rETRs*. In 4x plants, this effect was visible in PPFDs ≤ 50 µmol m^−2^ s^−1^, in 2x plants only at 10 µmol m^−1^ s^−2^.

#### 3.4.3. Kautsky Curce (KC) Experiment

**Figure 5:**
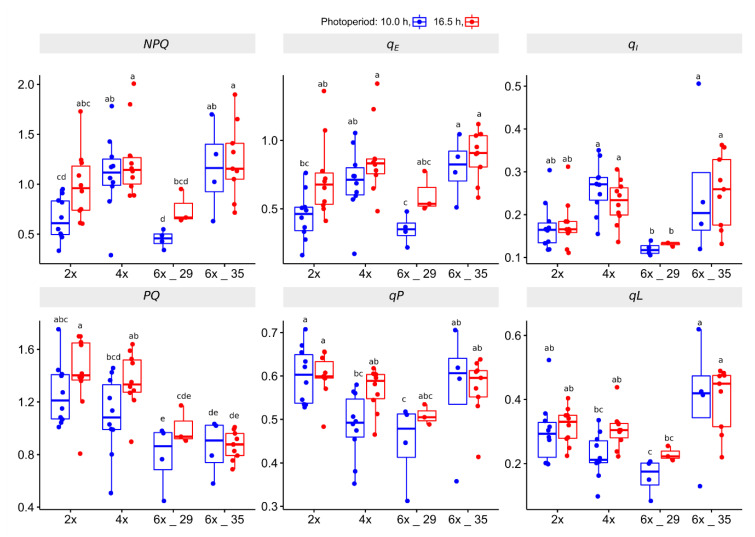
Coefficients calculated from Kautsky curve (KC) experiments of *R. auricomus* cytotypes exposed to two different photoperiods; non-photochemical quenching: NPQ, *qE* (heat dissipation NPQ), *qI* (photoinbitory NPQ); photochemical quenching: *PQ*, *qP*, *qL* (estimates of open non-reduced PSII centers). For summary statistics, see Table S6, letters represent 95% Duncan. Boxplots show the 25th, median, and 75th percentile range, and jitter plots represent the exact data distribution.

The Kautsky curve experiment offered insights into non-photochemical and photochemical quenching, NPQ and PQ, of the various ploidies and how elongation of photoperiod affected these processes. Figure 5 shows the most important coefficients.

In terms of NPQ efficiency, the 4x and the 6x_35 plants showed the highest *NPQ* values that differed significantly from the 2x and 6x_29 plants when exposed to the shorter photoperiod. Its extension increased the levels to those of the 4x and 6x_35 plants. Notably, photoperiod extension did not affect the latter. The *q_E_* coefficient quantifies the amount of heat dissipation. The boxplots of *q_E_* reflected those of *NPQ*. The *q_I_* coefficient, which quantifies the photoinhibitory NPQ, indicated that especially the 6x_29 plants and, to a lesser extent, the 2x plants, developed rather weak activities compared to the other cytotypes.

In terms of PQ efficiency, the *PQ* coefficient suggested a correlation with ploidy (lower values indicate higher efficiency). The *q_P_* and the *q_L_* coefficients quantify the number of reduceable (open) PSII reaction centers. They concur with *PQ*, with the exception that 6x_35 obviously quenched less efficiently than the other cytotypes, whereas the 6x_29 plants showed the best PQ values (lowest number of open reaction centers). Photoperiod extension reduced PQ in 2x, 4x, and 6x_29 plants, but not in 6x_35 plants.

## 4. Discussion

### 4.1. Ploidy and Photoperiod Did Not Affect Quantum Yield in General

Quantum yields can be measured in darkness-adapted plants, *ϕ_max_*, and light-adapted plants, *ϕ_PSII_*. The latter depends on the quality of the ambient light and thus provides a less comparable result than *ϕ_max_*. In this study, the different cytotypes gave similar results for both quantum yield types, the only exception being clone 6x_35 that showed remarkable reductions in both quantum yield types (Figure 1a,b). Quantum yield reduction is regarded as a general stress indicator [56]. Another study that compared *Allium oleraceum* L. cytotypes ≥ 4x [57] also found high similarities in their *ϕ_max_* values, as others did in a study on *Phragmites australis* (Cav.) Trin. ex Steud. cytotypes [58]. Likewise, *ϕ_max_* did not differ between 2x and 4x *Lilium* hybrids [59].

The photoperiod extension from 10.0 to 16.5 h caused no effect on *ϕ_PSII_* and *ϕ_max_*. There are fewer studies that focus on the effects of photoperiod on quantum yield compared to those that focus on spectral composition. Leonardos and coworkers explored the effect of different light sources with different wavelength compositions in short day and long day photoperiods on the development of *Chrysanthemum* plants [60]. In this study, the authors also compared high-pressure sodium lamps, similar to those that were used in this study, and found that photoperiod did not affect quantum yield that, in contrast to this study, was determined not by fluorescence but by CO_2_ gas exchange measurements. Accordingly, the lower quantum yield of 6x_35 (Figure 1) must have been caused by other factors.

### 4.2. Fast Fluorescence Transient Analyis (OJIP) Identified Stress in Cytotype 6x_35

The fast fluorescent transient analysis (OJIP), a highly detailed analysis of *ϕ_max_*, provides insights into energy fluxes and performance of PSII. In congruence with the quantum yield values, 2x, 4x, and 6x_29 did not differ in the calculable coefficients even when the photoperiod was extended. Only cytotype 6x_35 differed significantly (Figure 3), especially when the photoperiod was extended to 16.5 h. The increased absorption and trapped energy fluxes in the reaction centers (*ABS/RC*, *TR*_0_*/RC*) and the increased electron transport (*ET*_0_*/RC*) were reflected in higher dissipation energy fluxes (*DI*_0_*/RC*). These observations resembled those that are seen when plants are submitted to heat stress, temperatures ≥ 40 °C [43], or UV-C irradiation [61]. These effects were especially prominent in the 16.5 h photoperiod that aggravated the stress of the 6x_35 plants. Accordingly, the overall performance index *PI_ABS_* of 6x_35 plants decreased significantly.

### 4.3. Light Curve (LC) Experiments Showed That Increasing Actinic Light Caused 4x Cytotypes to Show Lower rETRs and 6x_35 to Resemble 2x and 6x_29 Cytotypes

The exploration of how quantum yield changes in the presence of increased actinic light is widely used to explore electron transport through PSII [41]. In this study, concerns about bad comparability do not apply because the investigated plants belong to a closely related species complex, in which similar pigment compositions and leaf structures can be assumed to prevail. A comparison of the *rETR* values (Figure 4) showed that the 6x_35 plants showed the lowest *rETR* values at lower PPFDs (≤100 µmol m^−2^ s^−1^). These observations can be explained by the stressed state of plants from this clone.

However, when PPFD increased further, the 4x plants yielded the lowest *rETR* values and the 6x_35 plants started to resemble the 2x and 6x_29 cytotypes more closely. Differences of the two photoperiods, which were clearly visible at PPFDs ≤ 20 µmol m^−2^ s^−1^, vanished at higher PPFDs. The stress of the 6x_35 cytotype that was observed in the OJIP analysis did not explain the effects that appeared in the LC experiment. Rather idiosyncratically, the *rETR* of the 6x_35 cytotype improved relatively with increasing PPFD. The OJIP experiment was carried out with dark-adapted plants and the LC experiment indicated that, rather idiosyncratically, increasing PPFD seemed to alleviate the stress of the 6x_35 plants.

Light period extension could have acted as a priming effect in 2x and 4x plants. At 10 µmol m^−2^ s^−1^, the 16.5 h-exposed plants showed higher *rETRs* than the 10.0 h plants. It disappeared, however, at PPFDs ≥ 20 µmol m^−2^ s^−1^, and was never visible in either of the 6x cytotypes, despite their pronounced differences in quantum yield. Optimization studies of light regimes for greenhouse-reared lettuce revealed that moderate extension of the light period may improve growth but daily received light amounts should not exceed certain dosages [62]. A certain priming potential was attributed to a combination of high light with high temperature in tomatoes [63]. These observations probably do not apply generally to all plant species. At least, albeit interpretation of the observed effects is difficult, subtle differences between the investigated cytotypes became evident.

Thus far, the discussed experiments did not include quenching mechanisms that have evolved to protect the photosynthesis in high-light conditions, NPQ and PQ [17,20]. The terms non-photochemical and photochemical are somewhat misleading because both of them involve chemical reactions. NPQ covers all quenching chemical reactions with the exception of the exciton trapping act, while PQ covers those affecting the exciton trapping act [64]. The Kautsky curve experiments (KC) informed about the quenching chemical reactions and represent the focus of the next section.

### 4.4. Kautsky Curve (KC) Analyses Identify Polploids as More Efficient Photosynthesis Quenchers

In this experiment, the actinic light was set to 300 µmol m^−2^ s^−1^, which represents the generally assumed saturation PPFD of photosynthesis in plants [65]. Its advantage is that it allows for exploring the quenching processes in the investigated plant leaves (Figure 5). In terms of NPQ, the polyploid 4x and 6x_35 cytotypes proved to be the most efficient ones. The coefficient *q_E_*, which quantifies heat dissipation, provided a highly similar picture to that of coefficient *NPQ*. The coefficient *q_I_*, which quantifies photoinhibitory NPQ, the minor component of NPQ, yielded similar results. NPQ seemed to increase in polyploids. Rakić and co-workers, who compared the diploid *Ramonda nathaliae* Panč et Petrov with the hexaploid *R. serbica* Panč in terms of photosynthesis after rewetting following a desiccation period, to which these rock-dwelling Gesneriaceae are exposed during the summer months, reported similar findings [66]. The hexaploid *R. serbica* showed higher NPQ levels than the diploid *R. nathaliae*. A study on artificial *Lilium* hybrids confirmed these observations [59]. The big differences in the coefficient values between the 6x_29 and 6x_35 cytotype may, at first glance, contradict the hypothesis that polyploids are more efficient in NPQ. In congruence with Achenbach and co-workers [58], ploidy alone does not suffice to explain photosynthesis efficiency data. If, however, the adaption history to forest habitats with a tree canopy and to open meadows is taken into account, the observed patterns make more sense, in which the open habitat-adapted 4x and 6x_35 plants represent the more efficient NPQ types and the forest-dwelling 2x and 6x_29 cytotypes the less efficient NPQ types.

PQ describes the chemistry at the PSII reaction centers. The coefficients *q_P_*, and even more so *q_L_*, are regarded as the most informative [67]. They quantify the number of open (unreduced) PSII reaction centers. The lower the number, the more efficient PQ becomes. By the majority, and as suggested by *q_P_* and *q_L_*, the polyploids were able to use PQ better than the diploids. The only exception is cytotype 6x_35, in which PQ is on the same level as in the diploids. The coefficient *PQ*, corresponding to *NPQ*, deviated by assigning similar efficiencies to 6x_29 and 6x_35, but is not considered as meaningful as *q_P_* and *q_L_*.

Light period extension only slightly increased the NPQ capabilities of the 2x cytotype. Generally, in terms of NPQ and PQ, polyploidy contributed more to differences than the light period extension that was applied in this study.

### 4.5. Photosynthesis, Habitat Adaptation, and Apomixis

This study utilized 2x, 4x, and 6x cytotypes from the *Ranunculus auricomus* species complex. Especially in the more sophisticated fluorescence experiments, which in terms of information quality exceeded simple quantum yield measurements of either light- or dark-adapted plants, the photosynthesis efficiency of polyploids was found to be better adapted in open habitat-conditioned plants, especially in the 4x cytotype.

The second open habitat cytotype, 6x_35, yielded rather controversial results. High value variations in photosynthesis efficiency of polyploids can occur as a result of higher intracytotype variation and niche breadth [57]. In terms of NPQ, 6x_35 resembled the 4x cytotype, in terms of PQ the 2x cytotype. The 6x_35 plants appeared to be more stressed when dark-adapted. With increasing actinic light, the quantum yield was again more similar for the 2x and 6x_29 cytotype and the 4x cytotype showed the lowest values, which may be due to the efficient quenching capabilities, as would be expected for open habitat-adapted species. The canopy-adapted 2x and 6x_29 cytotypes were less efficient in terms of NPQ. In terms of PQ, the 6x_29 followed the polyploid trend to be more efficient. The results of this study point more to NPQ than to PQ as an adaptive mechanism to open habitats. Probably, the success of a cytotype depends on the co-ordination extent of both quenching processes. Suboptimal co-ordination, as perhaps in 6x_35, may be responsible for the observed idiosyncratic results.

For the experiments in this study, the plants were grown under equal garden conditions, but different pre-adaptations from original habitats of the accessions may still have influenced their photosynthesis performance. Diploids originated from crosses of forest plants [38] and pre-adaptations to low-light conditions can be recognized in their photosynthesis. They responded to the extended photoperiod more intensively than the polyploids. Tetraploids were raised from light-adapted meadow plants, and the hexaploid clone 35 (=VRU2 in [57]) also originated from a typical open habitat-adapted mother plant from a meadow population with more sunlight exposure [38,68], whereas hexaploid clone 29 (=TRE in [57]) originated from a plant growing in a shaded habitat on a forest margin. Altogether, tetraploids appeared to be better adapted to high-light conditions by their efficient NPQ and PQ. Hexaploids, however, strongly differentiated according to their provenances, although 6x_35 behaved idiosyncratically, but not in terms of NPQ. All cytotypes originate from sites in Central Europe (at 47–48 ° latitude and from the same altitudinal zone), and hence we can rule out that differential light intensities due to strong latitude (or altitude) gradients, as observed in a study over the whole of Europe [30], would have influenced the evolution of their photosynthetic performance. The variability of polyploid ecotypes in *R. auricomus* may rely on a greater variance in gene expression patterns as they have highly heterozygous genomes [30]. In a comparison of diploid and tetraploid *Glycine max*, overexpression of oxidative stress-regulating genes correlated to differential photosynthetic performance and adaptation to higher light intensities [8]. Additionally, epigenetic control mechanisms may play a role. A study on cytosine methylation of diploid and tetraploid *R. kuepferi* revealed not only different methylation profiles between cytotypes, but also indicated two different epigenetic groups within tetraploids, correlating with different temperature exposures [69].

The photosynthesis performance of *R. auricomus* cytotypes, however, does relate to mode of ovule formation, as predominantly sexual diploids showed the highest sensitivity to an extended photoperiod and the lowest quenching capacities concomitant to the highest proportions of sexual ovules. In tetraploids, apomictic ovules prevailed [26]. Hexaploids, however, exhibited a very large variance in the proportions of sexual ovules, in congruence with observations on photosynthesis efficiency [26]. Variation was mostly attributed to two different ecotypes, one adapted to the canopy, the other to open habitats. We assume that differential levels of oxidative stress affect the mode of reproduction, similarly as has been suggested for different ploidy levels in the brassicacean genus *Boechera* [24].

## Figures and Tables

**Table 1:** Origin of 2x, 4x, and 6x *Ranunculus auricomus* plants.

Ploidy	Origin
2x	Synthetic F2 hybrid crosses of the sexual taxa *R. carpaticola* × *R. notabilis* that occur in forest sites in Central Europe [38].
4x	Open habitat-adapted meadow type grown from seeds of plants that were originally collected near Schönau, Mühlkreis, Austria (48°22′46.00″ N 14°44′46.00″ E, wet meadow) by L. Hodač and K. Spitzer (LH002).
6x	Hexaploid plants (6x) were grown from seeds of natural hybrids of *R. carpaticola* × *R. cassubicifolius* from Slovakia (original clone 29 from a forest margin and clone 35 from a meadow [25,38]). Originally, both clones were combined to obtain sufficient replicate numbers.

## Data Availability

The raw data are deposited at GöttingenResearchOnline (https://data.goettingen-research-online.de/dataset.xhtml?persistentId=doi:10.25625/S7RGG7, accessed on 8 July 2021).

## Supplementary Materials

The following are available online at https://www.mdpi.com/article/10.3390/biology10080811/s1, Figure S1, Exemplary Kautsky curve of KC experiments, indication of fluorescence parameters and coefficients; Table S1, Sample and clone codes of plant accessions that were used in the various photosynthesis fluorescence experiments; Table S2, Calculation and definition of photosynthesis coefficients; Table S3, Summary statistics and 95% Tukey multiple range test of *ϕ**_PSII_* and *ϕ**_MAX_* among cytotypes exposed to different photoperiods; Table S4, Summary statistics and 95% Duncan multiple range test of coefficients from transient fluorescence analyses (OJIP); Table S5; Summary statistics and 95% Duncan multiple range test of relative electron transport rates (*rETR*) among cytotypes; Table S6; Summary statistics and 95% Duncan multiple range test of coefficients calculated from Kautsky curve experiments.

## References

[1] Landis J.B., Soltis D.E., Li Z., Marx H.E., Barker M.S., Tank D.C., Soltis P.S. (2018). Impact of whole-genome duplication events on diversification rates in angiosperms. Am. J. Bot..

[2] van de Peer Y., Ashman T.-L., Soltis P.S., Soltis D.E. (2021). Polyploidy: An evolutionary and ecological force in stressful times. Plant Cell.

[3] Alix K., Gérard P.R., Schwarzacher T., Heslop-Harrison J.S.P. (2017). Polyploidy and interspecific hybridization: Partners for adaptation, speciation and evolution in plants. Ann. Bot..

[4] Rao S., Tian Y., Xia X., Li Y., Chen J. (2020). Chromosome doubling mediates superior drought tolerance in *Lycium ruthenicum* via abscisic acid signaling. Hortic. Res..

[5] Lourkisti R., Froelicher Y., Herbette S., Morillon R., Tomi F., Gibernau M., Giannettini J., Berti L., Santini J. (2020). Triploid Citrus genotypes have a better tolerance to natural chilling conditions of photosynthetic capacities and specific leaf volatile organic compounds. Front. Plant Sci..

[6] Coate J.E., Powell A.F., Owens T.G., Doyle J.J. (2013). Transgressive physiological and transcriptomic responses to light stress in allopolyploid *Glycine dolichocarpa* (Leguminosae). Heredity.

[7] Warner D.A., Edwards G.E. (1993). Effects of polyploidy on photosynthesis. Photosynth. Res..

[8] Coate J.E., Luciano A.K., Seralathan V., Minchew K.J., Owens T.G., Doyle J.J. (2012). Anatomical, biochemical, and photosynthetic responses to recent allopolyploidy in *Glycine dolichocarpa* (Fabaceae). Am. J. Bot..

[9] Münzbergová Z., Haisel D. (2019). Effects of polyploidization on the contents of photosynthetic pigments are largely population-specific. Photosynth. Res..

[10] Schoenfelder K.P., Fox D.T. (2015). The expanding implications of polyploidy. J. Cell Biol..

[11] Jeong S., Clark S.E. (2005). Photoperiod regulates flower meristem development in *Arabidopsis thaliana*. Genetics.

[12] Bauerle W.L., Oren R., Way D.A., Qian S.S., Stoy P.C., Thornton P.E., Bowden J.D., Hoffman F.M., Reynolds R.F. (2012). Photoperiodic regulation of the seasonal pattern of photosynthetic capacity and the implications for carbon cycling. PNAS.

[13] Kinoshita T., Kume A., Hanba Y.T. (2021). Seasonal variations in photosynthetic functions of the urban landscape tree species *Gingko biloba*: Photoperiod is a key trait. Trees.

[14] Wu Z., Skelvåg A.O., Baadshaug O.H. (2004). Quantification of photoperiodic effects on growth of *Phleum pratense*. Ann. Bot..

[15] Sulpice R., Flis A., Ivakov A.A., Apelt F., Krohn N., Encke B., Abel C., Feil R., Lunn J.E., Stitt M. (2014). *Arabidopsis* coordinates the diurnal regulation of carbon allocation and growth across a wide range of photoperiods. Mol. Plant.

[16] Webb A.A.R., Seki M., Satake A., Caldana C. (2019). Continuous dynamic adjustment of the plant circadian oscillator. Nat. Commun..

[17] Müller P., Li X.P., Niyogi K.K. (2001). Non-photochemical quenching. A response to excess light energy. Plant Physiol..

[18] Considine M.J., Foyer C.H. (2020). Oxygen and reactive oxygen species-dependent regulation of plant growth and development. Plant Physiol..

[19] Vass I. (2012). Molecular mechanisms of photodamage in the Photosystem II complex. Biochim. Biophys. Acta.

[20] Demmig-Adams B., Garab G., Adams W., Govindjee (2014). Non-Photochemical Quenching and Energy Dissipation in Plants, Algae and Cyanobacteria.

[21] Sato S., Kamiyama M., Iwata T., Makita N., Furukawa H., Ikeda H. (2006). Moderate increase of mean daily temperature adversely affects fruit set of *Lycopersicon esculentum* by disrupting specific physiological processes in male reproductive development. Ann. Bot..

[22] Ma X., Su Z., Ma H. (2020). Molecular genetic analyses of abiotic stress responses during plant reproductive development. J. Exp. Bot..

[23] Asker S.E., Jerling L. (1992). Apomixis in Plants.

[24] Mateo de Arias M., Gao L., Sherwood D.A., Dwivedi K.K., Price B.J., Jamison M., Kowallis B.M., Carman J.G. (2020). Whether gametophytes are reduced or unreduced in angiosperms might be determined metabolically. Genes.

[25] Klatt S., Hadacek F., Hodač L., Brinkmann G., Eilerts M., Hojsgaard D., Hörandl E. (2016). Photoperiod extension enhances sexual megaspore formation and triggers metabolic reprogramming in facultative apomictic *Ranunculus auricomus*. Front. Plant Sci..

[26] Ulum F.B., Costa Castro C., Hörandl E. (2020). Ploidy-dependent effects of light stress on the mode of reproduction in the *Ranunculus auricomus* complex (Ranunculaceae). Front. Plant Sci..

[27] Selva J.P., Zappacosta D., Carballo J., Rodrigo J.M., Bellido A., Gallo C.A., Gallardo J., Echenique V. (2020). Genes modulating the increase in sexuality in the facultative diplosporous grass *Eragrostis curvula* under water stress conditions. Genes.

[28] Karunarathne P., Reutemann A.V., Schedler M., Glücksberg A., Martínez E.J., Honfi A.I., Hojsgaard D.H. (2020). Sexual modulation in a polyploid grass: A reproductive contest between environmentally inducible sexual and genetically dominant apomictic pathways. Sci. Rep..

[29] Hörandl E., Hadacek F. (2013). The oxidative damage initiation hypothesis for meiosis. Plant Reprod..

[30] de Storme N., Geelen D. (2013). Sexual polyploidization in plants cytological mechanisms and molecular regulation. New Phytol..

[31] Barke B.H., Karbstein K., Daubert M., Hörandl E. (2020). The relation of meiotic behaviour to hybridity, polyploidy and apomixis in the Ranunculus auricomus complex (Ranunculaceae). BMC Plant Biol..

[32] Hojsgaard D., Greilhuber J., Pellino M., Paun O., Sharbel T.F., Hörandl E. (2014). Emergence of apospory and bypass of meiosis via apomixis after sexual hybridisation and polyploidisation. New Phytol..

[33] Pellino M., Hojsgaard D., Hörandl E., Sharbel T.F. (2020). Chasing the Apomictic Factors in the Ranunculus auricomus Complex: Exploring Gene Expression Patterns in Microdissected Sexual and Apomictic Ovules. Genes.

[34] Barke B.H., Daubert M., Hörandl E. (2018). Establishment of apomixis in diploid F2 hybrids and Inheritance of apospory from F1 to F2 hybrids of the *Ranunculus auricomus* complex. Front. Plant Sci..

[35] Nogler G.A. (1984). Genetics of apospory in apomictic *Ranunculus auricomus*. V: Conclusion. Bot. Helv..

[36] Hodač L., Scheben A.P., Hojsgaard D., Paun O., Hörandl E. (2014). ITS polymorphisms shed light on hybrid evolution in apomictic plants: A case study on the *Ranunculus auricomus* complex. PLoS ONE.

[37] Hörandl E., Dobeš C., Lambrou M. (1997). Chromosomen- und Pollenuntersuchungen an östereichischen Arten des apomiktischen *Ranunculus auricomus*-Komplexes. Bot. Helv..

[38] Hörandl E., Greilhuber J., Klimova K., Paun O., Temsch E., Emadzade K., Hodalova I. (2009). Reticulate evolution and taxonomic concepts in the *Ranunculus auricomus* complex (Ranunculaceae): Insights from analysis of morphological, karyological and molecular data. Taxon.

[39] Karbstein K., Tomasello S., Hodač L., Lorberg E., Daubert M., Hörandl E. (2021). Moving beyond assumptions: Polyploidy and environmental effects explain a geographical parthenogenesis scenario in European plants. Mol. Ecol..

[40] Pellino M., Hojsgaard D., Schmutzer T., Scholz U., Hörandl E., Vogel H., Sharbel T.F. (2013). Asexual genome evolution in the apomictic *Ranunculus auricomus* complex: Examining the effects of hybridization and mutation accumulation. Mol. Ecol..

[41] Baker N.R. (2008). Chlorophyll fluorescence: A probe of photosynthesis in vivo. Annu. Rev. Plant Biol..

[42] Murchie E.H., Lawson T. (2013). Chlorophyll fluorescence analysis: A guide to good practice and understanding some new applications. J. Exp. Bot..

[43] Strasser R.J., Srivastava A., Tsimilli-Michael M., Yunus M. (2000). The fluorescence transient as a tool to characterize and screen photosynthetic samples. Probing Photosynthesis: Mechanisms, Regulation, and Adaptation.

[44] Lazár D. (2015). Parameters of photosynthetic energy partitioning. J. Plant Physiol..

[45] Tsimilli-Michael M., Strasser R.J., Varma A. (2008). In vivo assessment of stress impact on plant’s vitality: Applications in detecting and evaluating the beneficial role of mycorrhization on host plants. Mycorrhiza: State of the Art, Genetics and Molecular Biology, Eco-Function, Biotechnology, Eco-Physiology, Structure and Systematics.

[46] Stirbet A., Lazár D., Kromdijk J., Govindjee (2018). Chlorophyll a fluorescence induction: Can just a one-second measurement be used to quantify abiotic stress responses?. Photosynthetica.

[47] Fiaz S., Wang X., Younas A., Alharthi B., Riaz A., Ali H. (2021). Apomixis and strategies to induce apomixis to preserve hybrid vigor for multiple generations. GM Crops Food.

[48] Schmidt A. (2020). Controlling apomixis: Shared features and distinct characteristics of gene regulation. Genes.

[49] Hodač L., Barke B.H., Hörandl E. (2018). Mendelian segregation of leaf phenotypes in experimental F 2 hybrids elucidates origin of morphological diversity of the apomictic *Ranunculus auricomus* complex. Taxon.

[50] R Core Team R: A Language and Environment for Statistical Computing. https://www.R-project.org/.

[51] Wickham H., François R., Lionel H., Müller K. (2021). Dplyr: A Grammar of Data Manipulation. https://CRAN.R-project.org/package=dplyr.

[52] Wickham H. (2021). Tidyr: Tidy Messy Data. https://CRAN.R-project.org/package=tidyr.

[53] Wickham H. (2016). Ggplot2: Elegant Graphics for Data Analysis.

[54] Kassambara A. (2020). Ggpubr: ‘Ggplot2’ Based Publication Ready Plots. https://CRAN.R-project.org/package=ggpubr.

[55] Brooks M.E., Kristensen K., van Benthem K.J., Magnusson A., Berg C.W., Nielsen A., Skaug H.J., Mächler M., Bolker B.M. (2017). glmmTMB balances speed and flexibility mong packages for zero-inflated generalized linear mixed modeling. R. J..

[56] Havaux M. (1992). Stress tolerance of photosystem ii in vivo: Antagonistic effects of water, heat, and photoinhibition stresses. Plant Physiol..

[57] Ježilová E., Nožková-Hlaváčková V., Duchoslav M. (2015). Photosynthetic characteristics of three ploidy levels of *Allium oleraceum* L. (Amaryllidaceae) differing in ecological amplitude. Plant Cell Environ..

[58] Achenbach L., Lambertini C., Brix H. (2012). Phenotypic traits of *Phragmites australis* clones are not related to ploidy level and distribution range. AoB Plants.

[59] Cao Q., Zhang X., Gao X., Wang L., Jia G. (2018). Effects of ploidy level on the cellular, photochemical and photosynthetic characteristics in *Lilium* FO hybrids. Plant Physiol. Biochem..

[60] Leonardos E.D., Ma X., Lanoue J., Grodzinski B. (2019). Leaf and whole-plant gas exchange and water-use efficiency of chrysanthemums under HPS and LEDs during the vegetative and flower-induction stages. Can. J. Plant Sci..

[61] Rusaczonek A., Czarnocka W., Kacprzak S., Witoń D., Ślesak I., Szechyńska-Hebda M., Gawroński P., Karpiński S. (2015). Role of phytochromes A and B in the regulation of cell death and acclimatory responses to UV stress in *Arabidopsis thaliana*. J. Exp. Bot..

[62] Weaver G., van Iersel M.W. (2020). Longer photoperiods with adaptive lighting control can improve growth of greenhouse-grown ‘Little Gem’ lettuce (*Lactuca sativa*). Hortscience.

[63] Zhou R., Yu X., Li X., Mendanha dos Santos T., Rosenqvist E., Ottosen C.-O. (2020). Combined high light and heat stress induced complex response in tomato with better leaf cooling after heat priming. Plant Physiol. Biochem..

[64] Papageorgiou G.C., Govindjee, Demmig-Adams B., Garab G., Adams W., Govindjee (2014). The non-photochemical quenching of the electronically excited state of chlorophyll a in plants: Definitions, timelines, viewpoints, open questions. Non-Photochemical Quenching and Energy Dissipation in Plants, Algae and Cyanobacteria.

[65] Ribeiro R.V., Machado E.C., de Oliveira R.F. (2006). Temperature response of photosynthesis and its interaction with light intensity in sweet orange leaf discs under non-photorespiratory condition. Ciênc. Agrotec..

[66] Rakić T., Gajić G., Lazarević M., Stevanović B. (2015). Effects of different light intensities, CO_2_ concentrations, temperatures and drought stress on photosynthetic activity in two paleoendemic resurrection plant species *Ramonda serbica* and *R. nathaliae*. Environ. Exp. Bot..

[67] Kramer D.M., Johnson G., Kiirats O., Edwards G.E. (2004). New fluorescence parameters for the determination of QA redox state and excitation energy fluxes. Photosynth. Res..

[68] Paun O., Greilhuber J., Temsch E.M., Hörandl E. (2006). Patterns, sources and ecological implications of clonal diversity in apomictic *Ranunculus carpaticola* (*Ranunculus auricomus* complex, Ranunculaceae). Mol. Ecol..

[69] Schinkel C.C.F., Syngelaki E., Kirchheimer B., Dullinger S., Klatt S., Hörandl E. (2020). Epigenetic patterns and geographical parthenogenesis in the alpine plant species *Ranunculus kuepferi* (Ranunculaceae). Int. J. Mol. Sci..

