# Supplementary material for: Polyploidy Improves Photosynthesis Regulation within the Ranunculus auricomus Complex (Ranunculaceae)
Source: Biology (Basel). 2021 Aug 21;10(8):811. doi: 10.3390/biology10080811 (PMC8389576; doi:10.3390/biology10080811)
Supplement: Supplementary file 1 [file biology-10-00811-s001.zip › biology-1312550-supplementary.pdf]

Table S1. Sample and clone codes of plant accessions that were used in the various photosynthesis fluorescence experiments

Sample	Plant ID	φ_{PSII}	LC	KC	OJIP
**2x, 10.0 h**					
C2-12	J10xJ30/03	x			
C2-13	J10xJ30/11	x	x	x	x
C2-15	J20xJ2/16	x	x	x	x
C2-19	J24xJ22/12	x	x	x	x
C2-21	F3xJ6/25	x	x	x	x
C2-24	J10xJ14/18	x		x	x
C2-25	J6xF3/19		x	x	x
C2-26	J10xJ30/05	x	x		
C2-27	J6xF3/14		x	x	x
C2-4	F3xJ6/28	x			
C2-7	J6xF3/14	x	x	x	x
C2-8	J6xF7/12	x	x	x	x
C2-9	J6xF7/14	x	x	x	x
**2x, 16.5 h**					
S2-1	F3xJ6/01	x	x	x	x
S2-18	J24xJ22/03	x	x	x	x
S2-2	F3xJ6/04	x	x	x	x
S2-21	F3xJ6/19	x	x	x	x
S2-23	F10xJ3/03	x	x	x	x
S2-24	J6xF3/06	x	x	x	x
S2-25	J10xJ14/09	x			
S2-27	J24xJ22/09	x			
S2-3	F3xJ6/05	x	x	x	x
S2-6	J6xF3/02	x	x	x	x
S2-7	J6xF3/05	x	x	x	x
S2-9	J6xF7/08	x	x	x	x
**4x, 10.0 h**					
C4-11	LH1406030B4-08	x	x	x	x
C4-13	LH1406030B4-16	x	x	x	x
C4-15	LH1406030B4-18	x	x	x	x
C4-19	LH1406030B5-07	x	x	x	x
C4-21	LH1406030B5-16	x	x	x	x
C4-22	LH1406030B5-17	x	x	x	x
C4-23	LH1406030B5-19	x	x	x	x
C4-26	LH4B005	x			
C4-5	LH1406030B2-04	x	x	x	x
C4-8	LH1406030B4-02	x	x	x	x
C4-9	LH1406030B4-05	x	x	x	x
**4x, 16.5 h**					
S4-11	LH1406030B4-19	x	x	x	x
S4-14	LH1406030B2-02	x	x	x	x
S4-16	LH1406030B5-05	x			x
S4-2	LH1406030B1-04	x		x	
S4-20	LH1406030B5-13	x			x
S4-21	LH1406030B5-18	x	x	x	
S4-22	LH1406030B5-20	x			
S4-23	LH1406030B4-10	x	x	x	x
S4-24	LH1406030G1-8	x			x
S4-25	LH1406030G1-16		x	x	x
S4-26	LH1406030G1-18		x	x	
S4-27	LH1406030B5-04	x			
S4-4	LH1406030B2-01	x			x
S4-5	LH1406030B2-07	x	x	x	
S4-6	LH1406030B4-01		x	x	x
S4-8	LH1406030B4-11	x	x	x	
**6x_29, 10.0 h**					
C6-1	29/15-3N/02	x	x		
C6-12	29/15-5K/21	x	x	x	x
C6-5	29/15-5K/02	x	x	x	x
C6-6	29/15-5K/05	x	x	x	x
C6-7	29/15-5K/09	x	x	x	x
**6x_29, 16.5 h**					
S6-39	29/15-5K/20	x	x	x	x
S6-9	29/15-5K/06	x	x	x	x
S6-1	29/15-5K/03	x	x	x	x
**6x_35, 10.0 h**					
C6-15	35/28-4*/26	x	x	x	x
C6-16	35/28-4*/28	x	x	x	x
C6-22	35/28-4a/16	x			x
C6-23	35/28-4*/27		x	x	x
C6-25	35/28-4a/22	x	x	x	x
C6-33	35/28-4Q/82	x	x		x
**6x_35, 16.5 h**					
S6-17	35/28-4*/03	x	x	x	x
S6-19	35/28-4*/18	x	x	x	x
S6-21	35/28-4*/24		x	x	x
S6-22	35/28-4*/22			x	
S6-24	35/28-4*/26		x	x	x
S6-25	35/28-4*/40		x	x	x
S6-29	35/28-4Q/27	x		x	
S6-33	35/28-4Q/28	x		x	
S6-37	35/28-4a/40			x	x

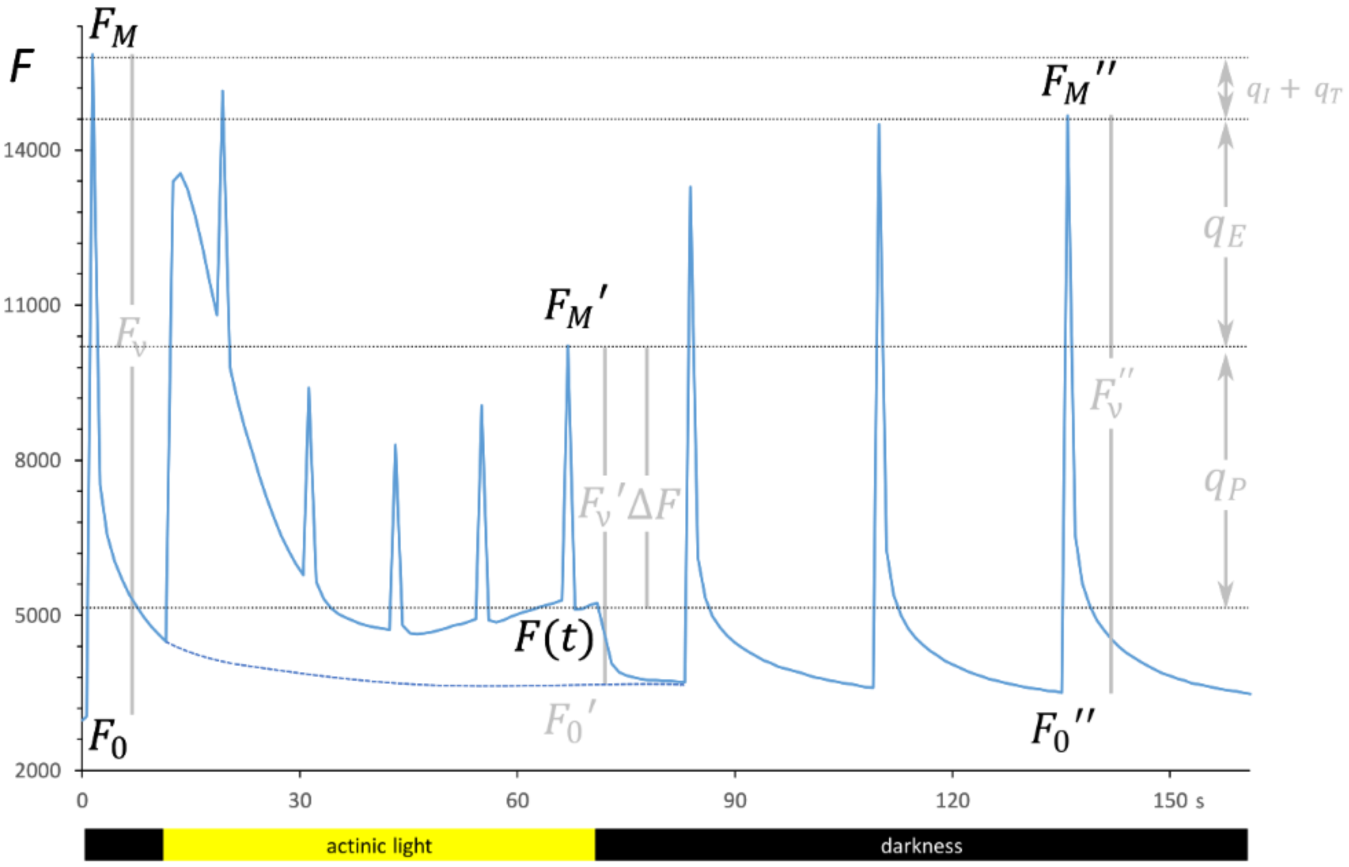

Figure S1: Exemplary Kautsky curve of KC experiments, indication of fluorescence parameters and coefficients, for details see Materials and Methods and Table S2.

Table S2. Calculation and definition of photosynthesis coefficients (Strasser and Govindjee, 1992; Strasser et al., 2004; Baker, 2008; Tsimilli-Michael and Strasser, 2013; Lazár, 2015; Rusaczonek et al., 2015)

Quenching of induced fluorescence analysis

F_0	Saturation pulse, baseline fluorescence
F_0'	Measuring pulse baseline fluorescence during actinic light phase
F_0''	Baseline fluorescence during subsequent dark phase
F_M	Saturation pulse, maximum fluorescence
F_M'	Saturation pulse, maximum fluorescence during actinic light phase
F_M''	Saturation pulse, maximum F during subsequent dark light phase
$F(t)$	Baseline F during actinic light phase
$F_V = F_M - F_0$	Variable F after adapted dark phase
$F_V' = F_M' - F_0'$	Variable F in actinic light phase
$F_V'' = F_M'' - F_0''$	Variable F in subsequent dark phase
$\Delta F = F_M' - F(t)$	Difference between F_V' and $F(t)$
$\phi_{max} = F_V/F_M$	PSII maximum quantum yield after dark adaptation
$\phi_{PSII} = F_V'/F_M'$	PSII potential quantum yield
$rETR = \phi_{PSII}$ x $PPFD$ x 0.5	Relative electron transport rate; PPFD: Photosynthetic photon flux density
$PQ = (F_M / F(t)) - (F_M / F_M')$	Photochemical quenching
$NPQ = (F_M / F_M') / F_M'$	Non-photochemical quenching
$q_E = (F_M'' - F_V') / F_M''$	Energy-dependent non-photochemical quenching
$q_I = 1 - (F_V'' / F_V)$	Photoinhibitory non-photochemical quenching coefficient
$q_P = \Delta F / (F_M' - F_0')$	Photochemical quenching coefficient, correlates nonlinearly to the fraction of "open" PSII centers
$q_L = ((F_M' - F(t)) / (F_M' - F_0'))$ x $(F_0' / F(t))$	Photochemical quenching coefficient, correlates linearly to the fraction of "open" PSII centers (lake model)

OJIP transient analysis

F_O $(= F_0)$	F intensity at 50 μs
F_J	F intensity at J-step (at 2ms)
F_I	F intensity at I-step (at 30 ms)
F_P $(= F_m)$	Maximum F intensity
$F_v = F_P - F_O$	Maximal variable F
F_P / F_O	F ratio
Area	Area between fluorescence curve and F_P
$M_0 = 4\ (F_{300\mu s} - F_O) / (F_M - F_O)$	Approximated initial slope of the *O–J* fluorescence transient (in ms^{-1})
$S_M = Area / (F_P - F_0)$	The normalized area below the *OJIP* curve until F_P
$V_I = (F_I - F_O) / (F_P - F_O)$	Relative fluorescence during the *I*-step
$V_J = (F_J - F_O) / (F_P - F_O)$	Relative fluorescence during the *J*-step
$N = S_M\ x\ M_0\ x\ (1 / V_J)$	Turn-over number of Q_A
$\phi_{Po} = F_V / F_P = \phi_{max}$	Maximum quantum yield of PSII
$V_I = (F_I - F_O) / (F_M - F_O)$	Relative variable fluorescence at the *I*-step
$V_J = (F_J - F_O) / (F_M - F_O)$	Relative variable fluorescence at the *J*-step
$\psi_0 = 1 - V_J$	Probability at t = 0 that an electron moves into the electron transport chain beyond Q_A^-

Specific energy fluxes

$ABS/RC = (M_0 / V_J) / \phi_{Po}$	Absorption flux per reaction center (RC) (apparent antenna size of an active PSII)
$TR_0/RC = M_0 / V_J$	Trapped energy flux per RC
ET_0/RC = (M_0 / V_j) x ψ_0	Electron transport flux per RC
DI_0/RC = ABS/RC – TR_0/RC	Dissipated energy flux per RC

Performance index

$PI_{ABS} = RC/ABS\ x\ (\phi_{Po} / 1 - \phi_{Po})\ x\ (\psi_0/1 - \psi_0)$	Performance index on absorption basis related to the overall photosynthetic activity of PSII

References:

Baker, N.R. (2008). Chlorophyll fluorescence: a probe of photosynthesis in vivo. *Annu. Rev. Plant Biol.* 59**,** 89-113.

Lazár, D. (2015). Parameters of photosynthetic energy partitioning. *Journal of Plant Physiology* 175, 131-147.

Rusaczonek, A., Czarnocka, W., Kacprzak, S., Witoń, D., Ślesak, I., Szechyńska-Hebda, M., et al. (2015). Role of phytochromes A and B in the regulation of cell death and acclimatory responses to UV stress in *Arabidopsis thaliana*. *Journal of Experimental Botany* 66(21)**,** 6679-6695.

Strasser, R.J., and Govindjee (1992). "The Fo and the OJIP Fluorescence Rise in Higher Plants and Algae," in *Regulation of Chloroplast Biogenesis,* ed. J.H. Argyroudi-Akoyunoglou. (Boston, MA: Springer US), 423-426.

Strasser, R.J., Tsimilli-Michael, M., and Srivastava, A. (2004). "Analysis of the chlorophyll a fluorescence transient," in *Chlorophyll a fluorescence*. Springer), 321-362.

Table S3. Summary statistics and 95% Tukey multiple range test of ϕ_{PSII} and ϕ_{MAX} among cytotypes exposed to different photoperiods (SE, standard error; CL, 95% confidential limits).

Parameter	Cytotype	Photoperiod (h)	Mean	SE	*n*	Lower CL	upper CL	Group
ϕ_{PSII}	**2x**	10.0	0.720	0.006	665	0.707	0.731	*b*
	**2x**	16.5	0.710	0.006	665	0.698	0.722	*b*
	**4x**	10.0	0.707	0.006	665	0.694	0.719	*b*
	**4x**	16.5	0.697	0.006	665	0.685	0.709	*ab*
	**6x_29**	10.0	0.720	0.010	665	0.699	0.739	*b*
	**6x_29**	16.5	0.722	0.012	665	0.698	0.745	*b*
	**6x_35**	10.0	0.617	0.010	665	0.597	0.636	*a*
	**6x_35**	16.5	0.676	0.009	665	0.659	0.693	*a*
ϕ_{MAX}	**2x**	10.0	0.821	0.008	170	0.804	0.837	*b*
	**2x**	16.5	0.815	0.009	170	0.797	0.832	*b*
	**4x**	10.0	0.823	0.009	170	0.805	0.839	*b*
	**4x**	16.5	0.818	0.008	170	0.802	0.833	*b*
	**6x_29**	10.0	0.826	0.012	170	0.800	0.849	*b*
	**6x_29**	16.5	0.808	0.017	170	0.774	0.839	*b*
	**6x_35**	10.0	0.705	0.015	170	0.674	0.735	*a*
	**6x_35**	16.5	0.671	0.014	170	0.643	0.697	*a*

Table S4. Summary statistic and 95% Duncan multiple range test of coefficients from transient fluorescence analyses (OJIP) of cytotypes that were exposed to two photoperiods (SD, standard deviation).

Coeff.	Cytotype	Photoper. (h)	Mean	SD	*n*	Min	Max	Group
ABS/RC	**2x**	10.0	1.902	0.181	10	1.662	2.264	*c*
	**2x**	16.5	2.090	0.154	10	1.926	2.400	*bc*
	**4x**	10.0	1.987	0.162	10	1.827	2.376	*c*
	**4x**	16.5	1.885	0.290	11	1.383	2.380	*c*
	**6x_29**	10.0	2.017	0.099	4	1.869	2.083	*c*
	**6x_29**	16.5	1.931	0.295	3	1.596	2.154	*c*
	**6x_35**	10.0	2.714	0.756	7	2.000	4.000	*b*
	**6x_35**	16.5	3.866	1.399	7	2.000	5.870	*a*
DI_0/RC	**2x**	10.0	0.336	0.052	10	0.268	0.412	*c*
	**2x**	16.5	0.403	0.054	10	0.350	0.498	*c*
	**4x**	10.0	0.357	0.045	10	0.313	0.460	*c*
	**4x**	16.5	0.354	0.087	11	0.224	0.485	*c*
	**6x_29**	10.0	0.357	0.028	4	0.319	0.384	*c*
	**6x_29**	16.5	0.350	0.078	3	0.260	0.395	*c*
	**6x_35**	10.0	0.867	0.551	7	0.348	2.000	*b*
	**6x_35**	16.5	1.545	0.905	7	0.504	2.974	*a*
TR_0/RC	**2x**	10.0	1.566	0.134	10	1.393	1.853	*c*
	**2x**	16.5	1.688	0.102	10	1.576	1.902	*c*
	**4x**	10.0	1.631	0.120	10	1.507	1.917	*c*
	**4x**	16.5	1.572	0.251	11	1.159	2.000	*c*
	**6x_29**	10.0	1.659	0.073	4	1.550	1.699	*c*
	**6x_29**	16.5	1.582	0.219	3	1.337	1.759	*c*
	**6x_35**	10.0	2.000	0.000	7	2.000	2.000	*b*
	**6x_35**	16.5	2.393	0.434	7	2.000	2.985	*a*
ET_0/RC	**2x**	10.0	0.964	0.067	10	0.866	1.097	*b*
	**2x**	16.5	0.993	0.047	10	0.917	1.072	*b*
	**4x**	10.0	0.981	0.057	10	0.887	1.047	*b*
	**4x**	16.5	0.886	0.081	11	0.699	0.985	*b*
	**6x_29**	10.0	0.965	0.071	4	0.910	1.067	*b*
	**6x_29**	16.5	0.952	0.079	3	0.862	1.011	*b*
	**6x_35**	10.0	0.987	0.023	7	0.942	1.000	*b*
	**6x_35**	16.5	1.301	0.331	7	0.949	1.794	*a*
PI_{ABS}	**2x**	10.0	4.121	1.014	10	2.666	5.928	*a*
	**2x**	16.5	3.005	0.786	10	1.986	3.997	*a*
	**4x**	10.0	3.696	1.044	10	2.073	4.965	*a*
	**4x**	16.5	3.990	2.502	11	1.000	9.670	*a*
	**6x_29**	10.0	3.267	0.650	4	2.547	3.919	*a*
	**6x_29**	16.5	3.909	1.715	3	2.622	5.856	*a*
	**6x_35**	10.0	1.410	0.949	7	0.325	3.000	*b*
	**6x_35**	16.5	0.787	0.645	7	0.224	2.000	*b*

Table S5. Summary statistics and 95% Duncan multiple range test of relative electron transport rates (*rETR*) among cytotypes that were exposed to different photoperiods in increasing PPFD intensities (SD, standard deviation).

PPFD (μmol m^{-2} sec^{-1})	Cytotype	Photoperiod (h)	Mean	SD	*n*	Min	Max	Group
10	**2x**	10.0	5.200	0.499	10	4.200	5.900	*bc*
	**2x**	16.5	5.720	0.361	10	5.300	6.400	*a*
	**4x**	10.0	4.860	0.556	10	4.000	5.700	*cd*
	**4x**	16.5	5.578	0.331	9	5.200	6.100	*ab*
	**6x_29**	10.0	5.160	0.288	5	4.900	5.600	*bc*
	**6x_29**	16.5	5.167	0.306	3	4.900	5.500	*bc*
	**6x_35**	10.0	4.500	0.339	5	4.100	4.900	*d*
	**6x_35**	16.5	4.460	0.416	5	4.000	4.900	*d*
20	**2x**	10.0	10.860	1.108	10	9.000	12.800	*ab*
	**2x**	16.5	11.640	0.595	10	10.800	12.800	*a*
	**4x**	10.0	10.440	0.947	10	9.000	12.200	*b*
	**4x**	16.5	11.622	0.484	9	10.800	12.400	*a*
	**6x_29**	10.0	10.720	0.502	5	10.400	11.600	*ab*
	**6x_29**	16.5	10.800	0.346	3	10.400	11.000	*ab*
	**6x_35**	10.0	9.040	0.669	5	8.400	10.000	*c*
	**6x_35**	16.5	9.120	0.944	5	8.000	10.200	*c*
50	**2x**	10.0	27.950	2.852	10	22.500	32.000	*ab*
	**2x**	16.5	29.500	1.683	10	27.000	32.500	*a*
	**4x**	10.0	26.600	2.492	10	23.000	31.000	*b*
	**4x**	16.5	29.000	1.031	9	27.500	31.000	*ab*
	**6x_29**	10.0	27.000	1.225	5	26.000	29.000	*ab*
	**6x_29**	16.5	27.833	0.764	3	27.000	28.500	*ab*
	**6x_35**	10.0	22.300	1.789	5	20.500	25.000	*c*
	**6x_35**	16.5	23.000	2.739	5	19.500	26.000	*c*
100	**2x**	10.0	49.500	6.786	10	36.000	58.000	*a*
	**2x**	16.5	52.000	4.422	10	44.000	59.000	*a*
	**4x**	10.0	47.000	5.121	10	39.000	55.000	*a*
	**4x**	16.5	51.000	2.550	9	47.000	55.000	*a*
	**6x_29**	10.0	47.600	1.673	5	45.000	49.000	*a*
	**6x_29**	16.5	49.333	1.528	3	48.000	51.000	*a*
	**6x_35**	10.0	39.200	2.683	5	37.000	43.000	*b*
	**6x_35**	16.5	41.000	5.339	5	35.000	47.000	*b*
300	**2x**	10.0	88.800	21.872	10	51.000	117.000	*ab*
	**2x**	16.5	90.900	13.932	10	57.000	102.000	*ab*
	**4x**	10.0	67.500	9.618	10	51.000	81.000	*c*
	**4x**	16.5	75.000	13.332	9	54.000	90.000	*bc*
	**6x_29**	10.0	88.800	9.149	5	78.000	99.000	*ab*
	**6x_29**	16.5	94.000	1.732	3	93.000	96.000	*abc*
	**6x_ 35**	10.0	70.200	6.573	5	60.000	78.000	*c*
	**6x_35**	16.5	76.200	11.345	5	63.000	87.000	*a*
500	**2x**	10.0	106.000	30.074	10	60.000	145.000	*ab*
	**2x**	16.5	103.500	18.265	10	60.000	120.000	*ab*
	**4x**	10.0	71.000	11.972	10	50.000	90.000	*d*
	**4x**	16.5	78.889	17.989	9	50.000	100.000	*cd*
	**6x_29**	10.0	113.000	12.550	5	100.000	125.000	*ab*
	**6x_29**	16.5	116.667	2.887	3	115.000	120.000	*a*
	**6x_35**	10.0	91.000	7.416	5	80.000	100.000	*bcd*
	**6x_35**	16.5	98.000	13.038	5	80.000	110.000	*abc*

Table S6. Summary statistic and 95% Duncan multiple range test of coefficients calculated from Kautsky curve experiments for different cytotypes that were exposed to two photoperiods (SD, standard deviation).

Coeff.	Cytotype	Photoper. (h)	Mean	SD	*n*	Min	Max	Group
NPQ	**2x**	10.0	0.654	0.213	10	0.334	0.951	*cd*
	**2x**	16.5	0.990	0.347	10	0.606	1.729	*abc*
	**4x**	10.0	1.102	0.390	10	0.290	1.782	*ab*
	**4x**	16.5	1.241	0.376	10	0.886	2.007	*a*
	**6x_29**	10.0	0.451	0.088	4	0.342	0.549	*d*
	**6x_29**	16.5	0.753	0.173	3	0.641	0.952	*bcd*
	**6x_35**	10.0	1.164	0.451	4	0.631	1.699	*ab*
	**6x_35**	16.5	1.237	0.381	9	0.716	1.897	*a*
q_E	**2x**	10.0	0.450	0.178	10	0.161	0.762	*bc*
	**2x**	16.5	0.728	0.289	10	0.412	1.361	*ab*
	**4x**	10.0	0.698	0.244	10	0.171	1.054	*ab*
	**4x**	16.5	0.869	0.269	10	0.483	1.415	*a*
	**6x_29**	10.0	0.350	0.108	4	0.218	0.480	*c*
	**6x_29**	16.5	0.605	0.149	3	0.503	0.776	*abc*
	**6x_35**	10.0	0.801	0.225	4	0.511	1.045	*a*
	**6x_35**	16.5	0.889	0.180	9	0.582	1.119	*a*
q_I	**2x**	10.0	0.172	0.057	10	0.118	0.304	*ab*
	**2x**	16.5	0.178	0.057	10	0.111	0.312	*ab*
	**4x**	10.0	0.263	0.060	10	0.155	0.350	*a*
	**4x**	16.5	0.229	0.052	10	0.136	0.306	*a*
	**6x_29**	10.0	0.120	0.015	4	0.105	0.139	*b*
	**6x_29**	16.5	0.131	0.005	3	0.126	0.134	*b*
	**6x_35**	10.0	0.258	0.171	4	0.120	0.506	*a*
	**6x_35**	16.5	0.256	0.085	9	0.132	0.363	*a*
PQ	**2x**	10.0	1.263	0.238	10	1.008	1.753	*abc*
	**2x**	16.5	1.425	0.276	10	0.808	1.700	*a*
	**4x**	10.0	1.093	0.296	10	0.507	1.458	*bcd*
	**4x**	16.5	1.358	0.218	10	0.897	1.639	*ab*
	**6x_29**	10.0	0.789	0.248	4	0.447	0.980	*e*
	**6x_29**	16.5	1.004	0.147	3	0.905	1.173	*cde*
	**6x_35**	10.0	0.856	0.214	4	0.580	1.032	*de*
	**6x_35**	16.5	0.869	0.111	9	0.688	1.008	*de*
q_P	**2x**	10.0	0.601	0.065	10	0.528	0.708	*a*
	**2x**	16.5	0.600	0.050	10	0.483	0.656	*a*
	**4x**	10.0	0.486	0.075	10	0.352	0.580	*bc*
	**4x**	16.5	0.569	0.049	10	0.465	0.618	*ab*
	**6x_29**	10.0	0.447	0.095	4	0.312	0.518	*c*
	**6x_29**	16.5	0.509	0.023	3	0.488	0.535	*abc*
	**6x_35**	10.0	0.569	0.149	4	0.358	0.706	*ab*
	**6x_35**	16.5	0.573	0.069	9	0.414	0.638	*ab*
q_L	**2x**	10.0	0.299	0.097	10	0.198	0.523	*ab*
	**2x**	16.5	0.317	0.057	10	0.224	0.404	*ab*
	**4x**	10.0	0.226	0.069	10	0.097	0.335	*bc*
	**4x**	16.5	0.306	0.059	10	0.222	0.438	*ab*
	**6x_29**	10.0	0.160	0.057	4	0.083	0.207	*c*
	**6x_29**	16.5	0.230	0.023	3	0.211	0.255	*bc*
	**6x_35**	10.0	0.397	0.202	4	0.129	0.619	*a*
	**6x_35**	16.5	0.402	0.101	9	0.220	0.489	*a*
